# Distinct Characteristics of Patients with Gout and an Underweight or Normal Body Mass Index: A Single-Center Retrospective Cross-Sectional Study

**DOI:** 10.3390/life15121876

**Published:** 2025-12-08

**Authors:** Sung Soo Ahn, Jiyoung Agatha Kim, Soorack Ryu, Yagop Shin, Sung Hoon Choi, Ka Young Choi, Kunhyung Bae

**Affiliations:** 1 Division of Rheumatology, Department of Internal Medicine, Yongin Severance Hospital, Yonsei University College of Medicine, Yongin 03722, Gyeonggi-do, Republic of Korea; 2 Department of Emergency Medicine, Seoul St. Mary’s Hospital, College of Medicine, The Catholic University of Korea, Seoul 06591, Republic of Korea; 3 Department of Medicine, Hanyang University College of Medicine, Seoul 04763, Republic of Korea; 4 Biostatistical Consulting and Research Lab, Medical Research Collaborating Center, Hanyang University, Seoul 04763, Republic of Korea; 5 Department of Orthopaedic Surgery, Hanyang University Hospital, Hanyang University College of Medicine, Seoul 04763, Republic of Korea; 6 Department of Anesthesiology and Pain Medicine, CHA Bundang Medical Center, CHA University School of Medicine, Seongnam 13488, Gyeonggi-do, Republic of Korea

**Keywords:** gout, body mass index, underweight, normal, obesity, overweight, predictor

## Abstract

Gout is an inflammatory arthritis triggered by monosodium urate crystal deposition, especially in obese patients. However, distinctions between the characteristics of obese and non-obese patients with gout remain unclear. We aimed to investigate the clinical differences by body mass index (BMI) with gout. We conducted a single-center retrospective cross-sectional study of 269 patients with gout from March 2020 to May 2024. Patients were classified into two groups: underweight/normal BMI and overweight/obesity. Baseline demographics, laboratory data, and clinical outcomes were compared between these groups. Stepwise logistic regression analysis was performed to identify predictors of underweight/normal BMI in gout patients. The underweight/normal BMI group included 35 patients (13.0%), characterized by older age, a higher proportion of females, and a lower prevalence of hypertension and alcohol consumption. This group also demonstrated lower uric acid, lipid profile, and alanine aminotransferase (ALT) levels but had a higher erythrocyte sedimentation rate. Logistic regression analysis identified female sex (odds ratio [OR] 3.831, 95% confidence interval [CI] 1.254–11.705, *p* = 0.018), presence of hypertension (OR 0.367, 95% CI 0.166–0.809, *p* = 0.013), total cholesterol (OR 0.990, 95% CI 0.982–0.999, *p* = 0.031), and ALT (OR 0.967, 95% CI 0.941–0.995, *p* = 0.019) as significant predictors of underweight/normal BMI gout. Understanding these characteristics may improve the identification of underweight/normal BMI subgroups, leading to improved approaches for gout management.

## 1. Introduction

Gout is an inflammatory arthritis caused by monosodium urate (MSU) crystal deposition, leading to acute and recurrent inflammation in affected joints and soft tissue [1,2]. While the first metatarsophalangeal joint is most commonly involved, gout can also affect the ankles, knees, elbows, wrists, and fingers [3]. Recurrent gout attacks can result in significant disability and may lead to destructive arthropathy [4]. Hyperuricemia—defined as serum uric acid levels of ≥7 mg/dL in males and ≥6 mg/dL in females—is a representative laboratory finding in patients with gout [5]. An interplay of environmental and genetic factors drives the elevation of uric acid levels in circulation, and subsequent MSU crystal formation, which are key events in gout pathogenesis [1,3,6]. The diagnosis typically requires the presence of suggestive symptoms or radiographic findings and elevated uric acid levels. With the global burden of gout rising, an estimated 95.8 million individuals may be affected by 2050 [7], prompting increased interest in characterizing patients with gout clinically.

Recently, there has been a remarkable increase in the obese population, affecting over 650 million adults worldwide [8]. In the United States alone, more than 42% of adults are now classified as having obesity [9]. The rise has significantly contributed to the prevalence of other metabolic disorders, including gout. Unbalanced dietary habits commonly found in obese individuals, such as the consumption of foods high in purines and fructose, have been identified as important contributors to hyperuricemia [10]. Furthermore, obesity itself is a risk factor for gout by promoting uric acid production and reducing renal excretion [11,12]. However, despite this strong correlation, gout is not limited to the obese population. Non-obese patients may develop gout through alternative pathogenic pathways, including genetic susceptibility, renal dysfunction, or other comorbid conditions [13,14,15]. Clinically, gout is often regarded as a disease of obesity, and the risk in non-obese individuals may be underestimated, potentially leading to delayed diagnosis or suboptimal management. Therefore, non-obese patients with gout may represent a distinct subgroup with different demographic or metabolic profiles. To the best of our knowledge, few studies have investigated their clinical characteristics. Therefore, we aimed to analyze differences in patient characteristics and clinical outcomes between underweight/normal body mass index (BMI) and overweight/obese patients. We hypothesized that gout patients with an underweight/normal BMI would exhibit a less pronounced metabolic syndrome profile and distinct demographic and laboratory outcomes.

## 2. Materials and Methods

### 2.1. Patient Selection

We conducted a retrospective cross-sectional study by analyzing the medical records of patients diagnosed with gout who visited the rheumatology clinic (either as outpatients or inpatients) at our hospital between March 2020 and May 2024. The diagnosis of gout was made according to the 1977 criteria of the American Rheumatism Association [16]. The inclusion criteria for this study were as follows: (i) first-time visitors to our rheumatology clinic, (ii) patients not currently taking uric acid-lowering medications, (iii) patients with a comprehensive medical history available, including BMI, current alcohol consumption, and smoking status, and (iv) patients with available routine laboratory results, including blood cell count and biochemical tests, as described in Section 2.2 (“Investigated variables”). The enrolled patients were categorized into two groups based on the WHO Asia–Pacific BMI classification, which defines 23 kg/m^2^ as the threshold for increased cardiometabolic risk in Asian populations and is widely applied in large epidemiologic studies and national screening programs in Republic of Korea [16,17].

### 2.2. Investigated Variables

Patient demographic data were collected at the initial visit, including age, sex, BMI, current alcohol consumption, smoking status, and diagnosis of new-onset disease (defined as disease onset within one month). Medical comorbidities were also noted, including hypertension, diabetes mellitus (DM), and dyslipidemia. Hypertension was identified by either the use of antihypertensive medications or a measured blood pressure of ≥140/90 mmHg, DM was determined by the use of anti-diabetic medications or a glycated hemoglobin (HbA1c) level of ≥6.5%, while dyslipidemia was diagnosed based on the use of anti-dyslipidemic medications or in accordance with the proposed criteria by the Korea National Health Screening Program [18]. For clinical outcomes, the incidence of gout flares and severe flares requiring hospitalization were investigated among those with one-year follow-up data.

All laboratory variables were measured using blood samples obtained at first hospital visit for diagnostic evaluation. Routine laboratory results included white blood cell (WBC) count, C-reactive protein (CRP), erythrocyte sedimentation rate (ESR), blood urea nitrogen (BUN), serum creatinine, serum uric acid, total cholesterol (TC), low-density lipoprotein cholesterol (LDL-C), high-density lipoprotein cholesterol (HDL-C), triglycerides, HbA1c, fasting glucose, aspartate aminotransferase (AST), and alanine aminotransferase (ALT).

### 2.3. Statistical Analysis

Continuous data were presented as means with standard deviations and categorical data as counts and percentages. Comparisons between the underweight/normal BMI and overweight/obesity groups were conducted using Student’s *t*-test, chi-square test, or Fisher’s exact test, as appropriate. Pearson or Spearman correlation analyses were used to evaluate the relationship between BMI and the investigated variables in each group. Furthermore, univariable and multivariable logistic regression analyses were performed using a stepwise variable selection method, with variables entered at *p* < 0.05 and removed at *p* < 0.05, thereby identifying the predictors associated with underweight/normal BMI in patients with gout. Additionally, receiver operating characteristic (ROC) analyses were performed to calculate the area under the curve (AUC) for classifying underweight/normal-BMI patients with gout. All statistical analyses were performed using IBM SPSS Statistics for Windows, version 26 (IBM Corp., Armonk, NY, USA), with a significance level set at a two-tailed *p*-value < 0.05.

## 3. Results

### 3.1. Comparison of Baseline Patient Characteristics Between the Two Groups

Among the 269 eligible patients with gout, 250 were male (92.9%) and 19 were female (7.1%). The mean age of the study population was 47.6 years, and the mean BMI was 27.4 kg/m^2^. Of these, 35 (13.0%) patients were grouped as the underweight/normal BMI group (BMI < 23 kg/m^2^) and 234 (87.0%) patients as the overweight/obesity group (BMI ≥ 23 kg/m^2^). Compared with the overweight/obesity group, the underweight/normal BMI group was older, included a higher proportion of females, and had lower rates of current alcohol consumption and hypertension. In addition, this group showed lower levels of uric acid, TC, LDL-C, triglycerides, and ALT but higher ESR levels (Table 1). Additionally, the comparison of patient groups based on the WHO BMI classification with a cutoff of 25 kg/m^2^ was presented in Supplementary Table S1.

Correlation analyses showed that, in the underweight/normal BMI group, only the female sex was significantly correlated with BMI (correlation coefficient: −0.337, 95% CI −0.603 to −0.004, *p* = 0.048). In contrast, in the overweight/obesity group, age (correlation coefficient: −0.287, 95% CI −0.400 to −0.165, *p* < 0.001), hypertension (correlation coefficient: 0.201, 95% CI 0.075 to 0.321, *p* = 0.002), ESR (correlation coefficient: −0.134, 95% CI −0.258 to −0.006, *p* = 0.040), and ALT (correlation coefficient: 0.314, 95% CI 0.194 to 0.425, *p* < 0.001) showed significant associations with BMI (Table 2).

### 3.2. Predictive Factors for Underweight/Normal BMI in Patients with Gout and Subgroup Analyses

**Figure 1:**
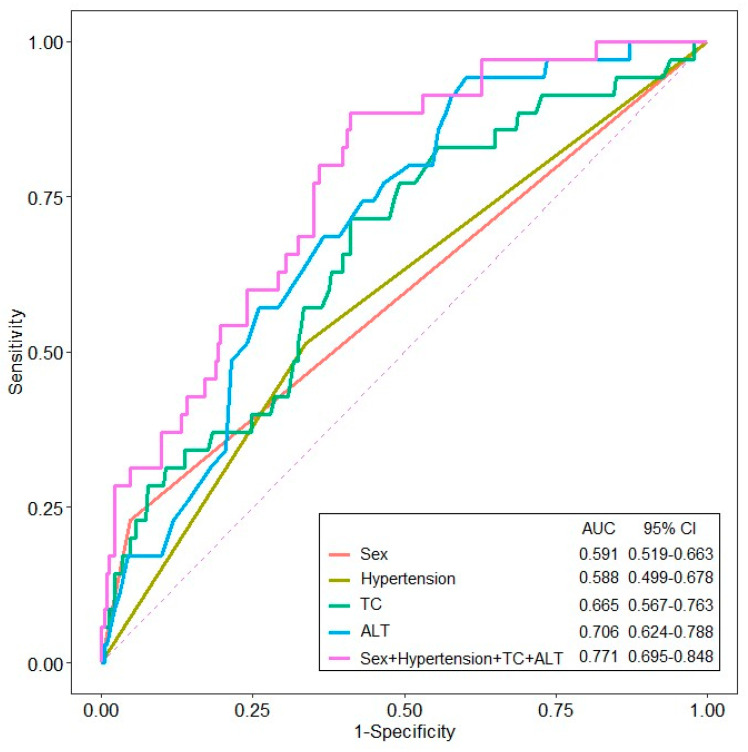
The receiver operating characteristic (ROC) curve of variables for predicting underweight/normal BMI patients with gout (ALT, alanine aminotransferase; TC, total cholesterol).

Logistic regression analysis identified female sex (OR = 3.831, 95% CI 1.254–11.705, *p* = 0.018), hypertension (OR = 0.367, 95% CI 0.166–0.809, *p* = 0.013), TC (OR = 0.990, 95% CI 0.982–0.999, *p* = 0.031), and ALT (OR = 0.967, 95% CI 0.941–0.995, *p* = 0.019) as factors associated with underweight/normal BMI (Table 3). The logistic regression analysis based on the WHO BMI classification of 25 kg/m^2^ was presented in Supplementary Table S2. ROC analysis of these factors revealed AUC values of 0.591 for females, 0.588 for those with a history of hypertension, 0.665 for TC, and 0.706 for ALT. A combined model of all four factors achieved an AUC of 0.771 (95% CI, 0.695–0.848) for distinguishing underweight/normal BMI from overweight/obese patients with gout (Figure 1).

Subgroup analyses were performed on a subset of patients with new-onset gout (*n* = 81) and male patients (*n* = 250). For new-onset patients, female sex (OR = 12.345, 95% CI 1.856–82.120, *p* = 0.009) and TC (OR = 0.964, 95% CI 0.942–0.987, *p* = 0.002) were predictive of underweight/normal BMI gout (Supplementary Table S3). Among male patients, LDL-C (OR = 0.989, 95% CI 0.978–0.999, *p* = 0.039) and ALT (OR = 0.966, 95% CI 0.938–0.995, *p* = 0.022) were associated with underweight/normal BMI in patients with gout (Supplementary Table S4).

### 3.3. Gout Flares After One-Year Follow-Up by BMI Status

At the one-year follow-up, no statistically significant differences were observed in gout flares (*p* = 0.755) or in flares requiring hospitalization (*p* = 0.686) between the underweight/normal BMI and overweight/obese patient groups (Supplementary Table S5).

## 4. Discussion

The global increase in the occurrence of gout and obesity, along with their close association, has resulted in these conditions becoming significant societal concerns. Notably, obesity and metabolic disorders are especially prevalent among patients with gout [19,20,21]. Nevertheless, the clinical characteristics of patients with non-obese gout remain poorly understood, and evaluating the clinical features of this disease subset could enhance the identification of these patients. In this study, comparison of baseline patient characteristics revealed that underweight/normal BMI patients were more likely to be older, included a higher proportion of females, and had lower rates of hypertension and alcohol consumption compared to overweight/obese patients. In addition, the underweight/normal BMI group had lower levels of uric acid, TC, LDL-C, triglycerides, and ALT, indicating distinct clinical characteristics. In the logistic regression analysis, female sex and the presence of hypertension, TC, and ALT were significant predictors of underweight/normal BMI. When these factors were integrated into the ROC analysis, the model achieved an AUC of 0.771, indicating fair diagnostic accuracy for underweight/normal BMI gout.

Obesity is traditionally considered a major risk factor for gout by increasing adipose tissue, which elevates uric acid levels in the circulation via enhanced production and diminished renal excretion [11,22]. Notably, our study revealed that 13.0% of patients were classified as underweight or normal BMI and showed distinct clinical characteristics compared with the overweight/obese group. These findings are in agreement with previous population-based studies reporting that gout prevalence was approximately 1–2% in normal-weight individuals and 4–7% in those with obesity [23]. These patients, who were more frequently elderly and female, showed lower prevalence of hypertension and reduced values of laboratory tests related to dyslipidemia, which are components of the metabolic syndrome. Specifically, the underweight/normal-BMI group showed lower lipid profiles and serum uric acid levels than overweight/obese patients (Table 1). In line with our findings, previous studies have reported that a lower BMI is associated with fewer metabolic abnormalities [24,25]. Furthermore, multivariate logistic regression analysis identified female sex, absence of hypertension, and lower TC and ALT levels as factors increasing the risk of being classified as underweight/normal BMI patient with gout. These findings emphasize that gout can also affect patients with lower BMI, even in the absence of traditional metabolic risk factors, and clinical vigilance by the attending physician is imperative for optimal diagnosis.

Our data revealed a significant correlation between sex and BMI in the underweight/normal-BMI group, a relationship not observed in other variables. In contrast, we observed that hypertension, dyslipidemia, uric acid, and ALT were positively correlated in the overweight/obese subgroup, which is consistent with typical gout-related characteristics. The inverse association between sex and BMI in the underweight/normal BMI subgroup could partly be attributed to the higher proportion of females, who generally have lower BMI than males [26]. However, the relatively small percentage of females (22.9%) in the underweight/normal BMI group suggests that additional factors likely contributed to the observed group differences. Indeed, in logistic regression analysis, hypertension, TC, and ALT decreased the risk of underweight/normal BMI gout. Similarly, in patients with new-onset disease, female sex and TC level were statistically significant predictors in logistic analysis. In an exclusive analysis of male patients, LDL-C and ALT levels were found to have an inverse relationship with underweight or normal BMI. Collectively, these findings suggest that patients with underweight/normal BMI with gout represent a distinct subgroup compared to patients with overweight/obesity, which likely reflects a different phenotype rather than a direct protective effect of these parameters on gout risk. From a pathophysiological aspect, lower TC and ALT in this subgroup may indicate a distinct metabolic condition characterized by reduced visceral adiposity and hepatic steatosis, in contrast to the typical phenotype of obesity-related “metabolic gout” [27,28]. Furthermore, older age, chronic inflammatory or catabolic states can be associated with both reduced serum cholesterol and impaired renal urate excretion, thereby predisposing to gout despite favorable lipid profiles [29,30]. In this context, low TC is more likely to indicate an alternative disease pathway than to act as a protective factor against gout itself. Although insulin resistance is a well-known risk factor for gout, our cross-sectional design could not directly assess this relationship. Nevertheless, the disparate metabolic pattern observed in our patients with underweight/normal BMI implies that, even in the absence of obesity or classical features of metabolic syndrome, subclinical insulin resistance or other non-obesity-related metabolic abnormalities may contribute to gout development [31,32]. Therefore, our findings indicate that gout should not be regarded only as a disease of patients with obesity and metabolic syndrome. Furthermore, genetic predisposition is also likely to contribute to gout development in patients with underweight/normal BMI. Genetic studies have identified urate transporter variants in genes such as ABCG2 and SLC2A9 as major determinants of serum urate levels and gout risk, with several risk alleles reported to be more prevalent in East Asian populations [33,34]. In this patient group, such genetic susceptibility may interact with modest environmental exposures to precipitate gout, while genetic testing for these variants is not possible in routine gout care. Accordingly, although our dataset did not include genetic information, future studies that integrate clinical, metabolic, and genomic data are warranted to clarify these gene–environment interactions.

In our cohort, underweight/normal-BMI patients with gout were more frequently female with lower ALT, TC, and hypertension rates than overweight/obese patients; however, these patients still developed gout. Thus, it is recommended for the clinicians be precautious to exclude gout solely based on BMI or the lack of typical metabolic syndrome features and diagnoses should be made with care in normal-BMI patients, particularly women, who present with compatible joint symptoms or hyperuricemia. Basic screening for blood pressure, lipid profile, and liver and renal function remains crucial in all patients with gout; however, in non-obese individuals, greater attention should be directed towards non–obesity-related factors such as renal urate handling, concomitant medications, and alcohol use. In addition, our study identified hypertension, lipid profile, and the liver enzyme ALT, along with female sex, as distinguishing factors between underweight/normal BMI and overweight/obese patients with gout. Intriguingly, ROC analysis using a model that included female sex, hypertension, TC, and ALT yielded an AUC of 0.771. This integrated model demonstrated higher diagnostic accuracy than individual variables alone, supporting its clinical utility in differentiating between underweight/normal BMI and overweight/obesity patients with gout [35]. Nevertheless, due to the modest performance of this model, further research is needed to precisely understand the diverse clinical aspects of gout.

Notably, some limitations should be considered when interpreting these findings. First, as a retrospective cross-sectional study, selection bias may have occurred, which could have influenced our results. Second, we did not include other relevant factors, such as genetic predisposition, intensity and frequency of exercise, or dietary intake. Third, BMI classification in this study was based on the WHO Asia–Pacific criteria, which divide patients into underweight/normal-BMI and overweight/obesity groups using a cutoff of 23 kg/m^2^. This threshold has been widely used in epidemiologic studies and clinical guidelines in Korea, as it better reflects cardiometabolic risk patterns in Asian populations. However, it differs from the global WHO classification. Because our study population consisted solely of Korean patients from a single medical center, the generalizability of our findings to other ethnic or regional populations may be limited. Fourth, because of the cross-sectional design, detailed information on gout history, including flare frequency, duration, and symptom severity, was unavailable. In addition, the gout phase at the time of blood sampling could not be standardized, as not all laboratory data were obtained at the identical phase of disease. Therefore, transient fluctuations in serum uric acid levels during acute attacks could not be completely excluded. Lastly, only 35 patients in our cohort had gout without obesity, and only 19 of them were female, representing a relatively small subgroup. Because of this limited sample size, the results of the multivariable analysis should be interpreted with caution, and some clinically important variables, such as serum uric acid, were not retained in the final model. Therefore, these findings should be considered exploratory, and further multicenter or nationwide cohort studies including a larger number of non-obese and female patients with gout are needed to validate and refine our understanding of this subgroup.

## 5. Conclusions

Our study demonstrates that underweight/normal-BMI patients with gout exhibit distinct clinical characteristics compared to overweight/obese patients. Female sex, hypertension, TC, and ALT levels were helpful in identifying this subgroup. Our findings suggest that the integration of these features may facilitate the recognition of a unique patient subpopulation with gout, contributing to a more nuanced understanding of the disease.

## Figures and Tables

**Table 1:** Patient characteristics of the underweight/normal BMI and overweight/obese groups.

	Total (*n* = 269)	Underweight/Normal BMI (*n* = 35)	Overweight/Obesity (*n* = 234)	*p*-Value
Demographic data				
Age (year)	**47.6 ± 17.2**	**56.6 ± 18.5**	**46.3 ± 16.6**	**<0.001**
Sex, female (%)	**19 (7.1)**	**8 (22.9)**	**11 (4.7)**	**<0.001**
BMI (kg/m^2^)	**27.4 ± 4.7**	**20.7 ± 2.4**	**28.4 ± 4.1**	**<0.001**
Alcohol (%)	**170 (63.2)**	**16 (45.7)**	**154 (65.8)**	**0.022**
Smoking (%)	95 (35.3)	12 (34.3)	83 (35.5)	0.891
New-onset disease (%)	81 (30.1)	10 (28.6)	71 (30.3)	0.832
Patient comorbidities				
Hypertension (%)	**172 (63.9)**	**17 (48.6)**	**155 (66.2)**	**0.043**
DM (%)	33 (12.3)	5 (14.3)	28 (12.0)	0.781
Dyslipidemia (%)	187 (69.5)	22 (62.9)	165 (70.5)	0.360
Laboratory data				
WBC count (cells/mm^3^)	7679.9 ± 2218.0	7321.7 ± 2171.2	7733.5 ± 2224.6	0.307
CRP (mg/L)	13.1 ± 28.4	19.3 ± 40.0	12.2 ± 26.2	0.169
ESR (mm/h)	**20.0 ± 24.4**	**31.2 ± 30.4**	**18.3 ± 22.9**	**0.003**
BUN (mg/dL)	15.3 ± 8.3	17.8 ± 10.9	14.9 ± 7.8	0.060
Creatinine (mg/dL)	1.0 ± 0.3	1.1 ± 0.5	1.0 ± 0.3	0.355
Uric acid (mg/dL)	**7.9 ± 1.7**	**7.2 ± 1.8**	**8.0 ± 1.6**	**0.008**
TC (mg/dL)	**186.1 ± 47.5**	**161.4 ± 51.5**	**189.8 ± 45.8**	**<0.001**
LDL-C (mg/dL)	**122.1 ± 40.7**	**101.8 ± 42.0**	**125.1 ± 39.7**	**0.002**
HDL-C (mg/dL)	46.2 ± 12.9	48.0 ± 18.8	46.0 ± 11.8	0.395
Triglyceride (mg/dL)	**182.6 ± 138.4**	**121.4 ± 83.7**	**191.7 ± 142.7**	**0.005**
HbA1c (%)	5.8 ± 0.8	5.8 ± 1.3	5.7 ± 0.7	0.667
Fasting glucose (mg/dL)	102.9 ± 23.5	101.1 ± 16.4	103.2 ± 24.4	0.628
AST (IU/L)	28.8 ± 27.2	23.6 ± 10.7	29.6 ± 28.8	0.228
ALT (IU/L)	**37.1 ± 31.7**	**21.4 ± 12.8**	**39.5 ± 33.0**	**0.002**

Medication use includes antihypertensive (*n* = 69, 40.1%), antidiabetic (*n* = 19, 11.0%), and lipid-lowering drugs (*n* = 58, 33.7%). Data are presented with mean ± standard deviation or number (%), as appropriate. Characteristics indicated in bold indicate statistical significance (*p* < 0.05). BMI, body mass index; DM, diabetes mellitus; WBC, white blood cell; CRP, C-reactive protein; ESR, erythrocyte sedimentation rate; BUN, blood urea nitrogen; TC, total cholesterol; LDL-C, low-density lipoprotein cholesterol; HDL-C, high-density lipoprotein cholesterol; HbA1c, glycated hemoglobin; AST, aspartate aminotransferase; ALT, alanine aminotransferase.

**Table 2:** Correlation between BMI and patient characteristics.

	Underweight/Normal BMI (*n* = 35)			Overweight/Obesity (*n* = 234)		
Characteristics	Correlation Coefficient	95% CI	*p*-Value	Correlation Coefficient	95% CI	*p*-Value
Age	−0.100	−0.419 to 0.241	0.568	−0.287	−0.400 to −0.165	<0.001
Sex, female	−0.337	−0.603 to −0.004	0.048	−0.020	−0.148 to 0.109	0.761
Alcohol	0.017	−0.318 to 0.348	0.923	−0.038	−0.165 to 0.091	0.568
Smoking	0.104	−0.237 to 0.423	0.551	−0.083	−0.209 to 0.045	0.203
New onset gout	−0.160	−0.468 to 0.183	0.359	−0.002	−0.130 to 0.126	0.977
Hypertension	0.133	−0.210 to 0.446	0.446	0.201	0.075 to 0.321	0.002
DM	0.057	−0.282 to 0.383	0.747	0.027	−0.102 to 0.155	0.680
Dyslipidemia	−0.100	−0.419 to 0.242	0.569	0.162	0.035 to 0.284	0.013
WBC count	−0.073	−0.397 to 0.266	0.676	0.022	−0.107 to 0.150	0.740
CRP	0.037	−0.300 to 0.365	0.834	−0.145	−0.268 to −0.017	0.027
ESR	−0.222	−0.517 to 0.120	0.200	−0.134	−0.258 to −0.006	0.040
BUN	−0.046	−0.374 to 0.291	0.791	−0.119	−0.243 to 0.010	0.071
Creatinine	−0.122	−0.438 to 0.220	0.484	−0.149	−0.272 to −0.021	0.023
Uric acid	−0.001	−0.334 to 0.332	0.994	0.190	0.063 to 0.311	0.004
TC	−0.003	−0.336 to 0.330	0.985	0.040	−0.088 to 0.168	0.540
LDL-C	−0.015	−0.346 to 0.320	0.934	0.089	−0.039 to 0.215	0.173
HDL-C	−0.010	−0.343 to 0.324	0.952	−0.122	−0.246 to 0.007	0.064
Triglyceride	−0.028	−0.358 to 0.308	0.871	0.035	−0.094 to 0.162	0.599
HbA1c	0.035	−0.301 to 0.364	0.840	0.101	−0.027 to 0.227	0.122
Fasting glucose	−0.021	−0.351 to 0.315	0.907	−0.027	−0.155 to 0.102	0.682
AST	−0.177	−0.482 to 0.166	0.310	0.010	−0.119 to 0.138	0.881
ALT	0.232	−0.110 to 0.525	0.180	0.314	0.194 to 0.425	<0.001

BMI, body mass index; CI, confidence interval; DM, diabetes mellitus; WBC, white blood cell; CRP, C-reactive protein; ESR, erythrocyte sedimentation rate; BUN, blood urea nitrogen; TC, total cholesterol; LDL-C, low-density lipoprotein cholesterol; HDL-C, high-density lipoprotein cholesterol; HbA1c, glycated hemoglobin; AST, aspartate aminotransferase; ALT, alanine aminotransferase.

**Table 3:** Logistic regression analysis of patient characteristics of underweight/normal BMI gout.

Characteristics	Univariate Analysis			Multivariate Analysis (Stepwise)		
	OR	95% CI	*p*-Value	OR	95% CI	*p*-Value
Age	1.032	1.012 to 1.053	0.001			
Sex, female	6.007	2.222 to 16.237	<0.001	3.831	1.254 to 11.705	0.018
Alcohol	0.438	0.213 to 0.897	0.024			
Smoking	0.949	0.449 to 2.005	0.891			
New onset gout	0.918	0.419 to 2.012	0.831			
Hypertension	0.481	0.235 to 0.985	0.045	0.367	0.166 to 0.809	0.013
DM	1.226	0.440 to 3.420	0.697			
Dyslipidemia	0.708	0.337 to 1.485	0.360			
WBC count	1.000	1.000 to 1.000	0.306			
CRP	1.007	0.997 to 1.017	0.183			
ESR	1.018	1.005 to 1.030	0.005			
BUN	1.031	0.997 to 1.066	0.076			
Creatinine	1.504	0.627 to 3.607	0.361			
Uric acid	0.738	0.589 to 0.924	0.008			
TC	0.987	0.979 to 0.995	0.001	0.990	0.982 to 0.999	0.031
LDL-C	0.986	0.977 to 0.995	0.002			
HDL-C	1.011	0.985 to 1.038	0.394			
Triglyceride	0.990	0.984 to 0.996	0.001			
HbA1c	1.093	0.730 to 1.635	0.667			
Fasting glucose	0.995	0.977 to 1.014	0.627			
AST	0.977	0.946 to 1.010	0.170			
ALT	0.953	0.926 to 0.981	0.001	0.967	0.941 to 0.995	0.019

BMI, body mass index; OR, odds ratio; CI, confidence interval; DM, diabetes mellitus; WBC, white blood cell; CRP, C-reactive protein; ESR, erythrocyte sedimentation rate; BUN, blood urea nitrogen; TC, total cholesterol; LDL-C, low-density lipoprotein cholesterol; HDL-C, high-density lipoprotein cholesterol; HbA1c, glycated hemoglobin; AST, aspartate aminotransferase; ALT, alanine aminotransferase.

## Data Availability

The datasets used and/or analyzed during the current study available from the corresponding author on reasonable request.

## Supplementary Materials

The following supporting information can be downloaded at: https://www.mdpi.com/article/10.3390/life15121876/s1, Table S1: Patient characteristics between the underweight/normal BMI and overweight/obesity groups by the standard WHO classification; Table S2: Logistic regression analysis of patient characteristics of underweight/normal BMI gout by the standard WHO classification; Table S3: Logistic regression analysis of predictive variables for gout in underweight/normal BMI patients with new-onset disease (*n* = 81); Table S4: Logistic regression analysis of the predictive variables for underweight/normal BMI gout in male patients (*n* = 250); Table S5: Disease outcomes during 1-year follow-up (*n* = 98).

## Abbreviations

The following abbreviations are used in this manuscript:

BMI	Body mass index
DM	Diabetes mellitus
WBC	White blood cell
CRP	C-reactive protein
ESR	Erythrocyte sedimentation rate
BUN	Blood urea nitrogen
TC	Total cholesterol
LDL-C	Low-density lipoprotein cholesterol
HDL-C	High-density lipoprotein cholesterol
HbA1c	Glycated hemoglobin
AST	Aspartate aminotransferase
ALT	Alanine aminotransferase
MSU	Monosodium urate
AUC	Area under the curve
ROC	Receiver operating characteristic
