# Supplementary material for: Distinct Characteristics of Patients with Gout and an Underweight or Normal Body Mass Index: A Single-Center Retrospective Cross-Sectional Study
Source: Life (Basel). 2025 Dec 8;15(12):1876. doi: 10.3390/life15121876 (PMC12734147; doi:10.3390/life15121876)
Supplement: Supplementary file 1 [file life-15-01876-s001.zip › life-3966900-supplementary.pdf]

Supplementary Table S1. Patient characteristics between the underweight/normal BMI and overweight/obesity groups by the standard WHO classification

	Total (n=269)	Underweight/normal BMI (n= 82)	Overweight/obesity (n= 187)	p-value
Demographic data				
**Age (year)**	**47.6 ± 17.2**	**53.2 ± 17.7**	**45.2 ± 16.4**	**<0.001**
**Sex, female (%)**	**19 (7.1)**	**10 (12.2)**	**9 (4.8)**	**0.030**
**BMI (kg/m²)**	**27.4 ± 4.7**	**22.6 ± 2.3**	**29.6 ± 3.9**	**<0.001**
Alcohol (%)	170 (63.2)	49 (59.8)	121 (64.7)	0.439
Smoking (%)	95 (35.3)	30 (36.6)	65 (34.8)	0.773
New-onset disease (%)	81 (30.1)	22 (26.8)	59 (31.6)	0.438
Patient comorbidities				
**Hypertension (%)**	**172 (63.9)**	**44 (53.7)**	**128 (68.4)**	**0.020**
DM (%)	33 (12.3)	9 (11.0)	24 (12.8)	0.669
Dyslipidemia (%)	187 (69.5)	49 (59.8)	138 (73.8)	0.022
Laboratory data				
WBC count (/mm³)	7679.9 ± 2218.0	7373.7 ± 2232.5	7814.2 ± 2204.3	0.134
**CRP (mg/L)**	**13.1 ± 28.4**	**19.0 ± 36.5**	**10.5 ± 23.7**	**0.024**
**ESR (mm/hr)**	**20.0 ± 24.4**	**26.9 ± 29.5**	**17.0 ± 21.1**	**0.002**
BUN (mg/dL)	15.3 ± 8.3	15.8 ± 8.2	15.1 ± 8.4	0.522
Creatinine (mg/dL)	1.0 ± 0.3	1.1 ± 0.4	1.0 ± 0.3	0.365
**Uric acid (mg/dL)**	**7.9 ± 1.7**	**7.5 ± 1.7**	**8.1 ± 1.6**	**0.004**
**TC (mg/dL)**	**186.1 ± 47.5**	**174.0 ± 48.7**	**191.4 ± 46.0**	**0.005**
**LDL-C (mg/dL)**	**122.1 ± 40.7**	**111.3 ± 38.2**	**126.8 ± 41.0**	**0.004**
HDL-C (mg/dL)	46.2 ± 12.9	47.5 ± 15.3	45.7 ± 11.7	0.284
**Triglyceride (mg/dL)**	**182.6 ± 138.4**	**149.9 ± 124.7**	**196.9 ± 142.0**	**0.010**
HbA1c (%)	5.8 ± 0.8	5.7 ± 1.0	5.8 ± 0.7	0.503
Fasting glucose (mg/dL)	102.9 ± 23.5	101.7 ± 16.9	103.4 ± 25.9	0.595
AST (IU/L)	28.8 ± 27.2	30.6 ± 42.1	28.0 ± 17.0	0.467
**ALT (IU/L)**	**37.1 ± 31.7**	**26.0 ± 18.5**	**42.0 ± 34.9**	**<0.001**

Data are presented with mean ± standard deviation or number (%), as appropriate

Characteristics indicated in bold indicate statistical significance ($p < 0.05$)

BMI, body mass index; DM, diabetes mellitus; WBC, white blood cell; CRP, C-reactive protein; ESR, erythrocyte sedimentation rate; BUN, blood urea nitrogen; TC, total cholesterol; LDL-C, low-density lipoprotein cholesterol; HDL-C, high-density lipoprotein cholesterol; HbA1c, glycated hemoglobin; AST, aspartate aminotransferase; ALT, alanine aminotransferase.

Supplementary Table S2. Logistic regression analysis of patient characteristics of underweight/normal BMI gout by the standard WHO classification

Characteristics	Univariate analysis			Multivariate analysis (Stepwise)		
	OR	95% CI	p-value	OR	95% CI	p-value
Age	1.027	1.011 to 1.043	<0.001	1.032	1.013 to 1.052	0.001
Sex, female	2.747	1.072 to 7.041	0.035			
Alcohol	0.810	0.475 to 1.381	0.439			
Smoking	1.083	0.631 to 1.860	0.773			
New-onset gout	0.796	0.446 to 1.418	0.438			
Hypertension	0.534	0.313 to 0.909	0.021	0.371	0.199 to 0.691	0.002
DM	0.837	0.371 to 1.890	0.669			
Dyslipidemia	0.527	0.305 to 0.913	0.022	0.504	0.275 to 0.923	0.027
WBC count	1.000	1.000 to 1.000	0.135			
CRP	1.010	1.001 to 1.019	0.036			
ESR	1.016	1.005 to 1.026	0.003			
BUN	1.010	0.980 to 1.041	0.523			
Creatinine	1.395	0.674 to 2.888	0.370			
Uric acid	0.784	0.664 to 0.927	0.004			
TC	0.992	0.987 to 0.998	0.006			
LDL-C	0.991	0.984 to 0.997	0.005			
HDL-C	1.011	0.991 to 1.031	0.284			
Triglyceride	0.996	0.993 to 0.999	0.009			
HbA1c	0.885	0.619 to 1.266	0.504			
Fasting glucose	0.997	0.985 to 1.009	0.596			
AST	1.003	0.994 to 1.012	0.476			
ALT	0.973	0.959 to 0.988	<0.001	0.980	0.966 to 0.996	0.011

BMI, body mass index; OR, odds ratio; CI, confidence interval; DM, diabetes mellitus; WBC, white blood cell; CRP, C-reactive protein; ESR, erythrocyte sedimentation rate; BUN, blood urea nitrogen; TC, total cholesterol; LDL-C, low-density lipoprotein cholesterol; HDL-C, high-density lipoprotein cholesterol; HbA1c, glycated hemoglobin; AST, aspartate aminotransferase; ALT, alanine aminotransferase.

Supplementary Table S3. Logistic regression analysis of predictive variables for gout in underweight/normal BMI patients with new-onset disease (n=81)

Characteristics	Univariate analysis			Multivariate analysis (Stepwise)		
	OR	95% CI	p-value	OR	95% CI	p-value
Age	1.050	1.015–1.087	0.005			
Sex, female	16.250	3.566 to 74.046	<0.001	12.345	1.856–82.120	0.009
Alcohol	0.233	0.055–0.981	0.047			
Smoking	0.218	0.026–1.820	0.159			
Hypertension	0.614	0.162–2.318	0.471			
DM	2.286	0.403–12.956	0.350			
Dyslipidemia	0.588	0.150–2.307	0.447			
WBC count	1.000	1.000–1.000	0.418			
CRP	1.022	1.001–1.042	0.036			
ESR	1.033	1.009–1.058	0.008			
BUN	1.027	0.985–1.071	0.211			
Creatinine	1.302	0.402–4.224	0.660			
Uric acid	0.488	0.289–0.825	0.007			
TC	0.962	0.942–0.982	<0.001	0.964	0.942–0.987	0.002
LDL-C	0.956	0.931–0.981	0.001			
HDL-C	1.004	0.955–1.056	0.875			
Triglyceride	0.967	0.948–0.987	0.001			
HbA1c	0.980	0.332–2.893	0.970			
Fasting glucose	0.991	0.955–1.028	0.633			
AST	0.955	0.881–1.035	0.264			
ALT	0.916	0.850–0.988	0.023			

BMI, body mass index; OR, odds ratio; CI, confidence interval; DM, diabetes mellitus; WBC, white blood cell; CRP, C-reactive protein; ESR, erythrocyte sedimentation rate; BUN, blood urea nitrogen; TC, total cholesterol; LDL-C, low-density lipoprotein cholesterol; HDL-C, high-density lipoprotein cholesterol; HbA1c, glycated hemoglobin; AST, aspartate aminotransferase; ALT, alanine aminotransferase.

Supplementary Table S4. Logistic regression analysis of the predictive variables for underweight/normal BMI gout in male patients (n=250)

Characteristics	Univariate analysis			Multivariate analysis (Stepwise)		
	OR	95% CI	p-value	OR	95% CI	p-value
Age	1.031	1.007–1.056	0.011			
Alcohol	0.596	0.265–1.339	0.210			
Smoking	1.430	0.638–3.205	0.385			
New onset gout	0.423	0.141–1.270	0.125			
Hypertension	0.579	0.259–1.294	0.183			
DM	1.377	0.440–4.308	0.582			
Dyslipidemia	0.625	0.275–1.417	0.260			
WBC count	1.000	1.000–1.000	0.577			
CRP	1.004	0.990–1.018	0.562			
ESR	1.013	0.998–1.028	0.099			
BUN	1.021	0.980–1.064	0.311			
Creatinine	1.065	0.322–3.521	0.918			
Uric acid	0.793	0.615–1.021	0.073			
TC	0.988	0.979–0.997	0.010			
LDL-C	0.986	0.976–0.997	0.010	0.989	0.978–0.999	0.039
HDL-C	1.012	0.982–1.044	0.427			
Triglyceride	0.992	0.986–0.998	0.015			
HbA1c	1.290	0.884–1.881	0.187			
Fasting glucose	1.000	0.983–1.017	1.000			
AST	0.976	0.940–1.013	0.202			
ALT	0.960	0.932–0.990	0.009	0.966	0.938–0.995	0.022

BMI, body mass index; OR, odds ratio; CI, confidence interval; DM, diabetes mellitus; WBC, white blood cell; CRP, C-reactive protein; ESR, erythrocyte sedimentation rate; BUN, blood urea nitrogen; TC, total cholesterol; LDL-C, low-density lipoprotein cholesterol; HDL-C, high-density lipoprotein cholesterol; HbA1c, glycated hemoglobin; AST, aspartate aminotransferase; ALT, alanine aminotransferase.

Supplementary Table S5. Disease outcomes during 1-year follow-up (n=98)

	Total (n=98)	Underweight/normal BMI (n= 11)	Overweight/obesity (n= 87)	p-value
Disease outcomes				
Disease flare	40 (40.8)	5 (45.5)	35 (40.2)	0.755
Flare requiring hospital care	19 (19.4)	1 (9.1)	18 (20.7)	0.686

Data are presented as number (%).

BMI, body mass index.
